# Analysis of the genetic diversity of influenza A viruses using next-generation DNA sequencing

**DOI:** 10.1186/s12864-015-1284-z

**Published:** 2015-02-14

**Authors:** Silvie Van den Hoecke, Judith Verhelst, Marnik Vuylsteke, Xavier Saelens

**Affiliations:** Department of Medical Protein Research, VIB, B-9052 Ghent, Belgium; Department of Biomedical Molecular Biology, Ghent University, B-9052 Ghent, Belgium; Gnomixx, Onafhankelijkheidslaan 38, B-9000 Ghent, Belgium

**Keywords:** Influenza virus, Quasispecies, Next-generation sequencing, Illumina MiSeq, Ion Torrent PGM, RT-PCR

## Abstract

**Background:**

Influenza viruses exist as a large group of closely related viral genomes, also called quasispecies. The composition of this influenza viral quasispecies can be determined by an accurate and sensitive sequencing technique and data analysis pipeline. We compared the suitability of two benchtop next-generation sequencers for whole genome influenza A quasispecies analysis: the Illumina MiSeq sequencing-by-synthesis and the Ion Torrent PGM semiconductor sequencing technique.

**Results:**

We first compared the accuracy and sensitivity of both sequencers using plasmid DNA and different ratios of wild type and mutant plasmid. Illumina MiSeq sequencing reads were one and a half times more accurate than those of the Ion Torrent PGM. The majority of sequencing errors were substitutions on the Illumina MiSeq and insertions and deletions, mostly in homopolymer regions, on the Ion Torrent PGM. To evaluate the suitability of the two techniques for determining the genome diversity of influenza A virus, we generated plasmid-derived PR8 virus and grew this virus *in vitro*. We also optimized an RT-PCR protocol to obtain uniform coverage of all eight genomic RNA segments. The sequencing reads obtained with both sequencers could successfully be assembled *de novo* into the segmented influenza virus genome. After mapping of the reads to the reference genome, we found that the detection limit for reliable recognition of variants in the viral genome required a frequency of 0.5% or higher. This threshold exceeds the background error rate resulting from the RT-PCR reaction and the sequencing method. Most of the variants in the PR8 virus genome were present in hemagglutinin, and these mutations were detected by both sequencers.

**Conclusions:**

Our approach underlines the power and limitations of two commonly used next-generation sequencers for the analysis of influenza virus gene diversity. We conclude that the Illumina MiSeq platform is better suited for detecting variant sequences whereas the Ion Torrent PGM platform has a shorter turnaround time. The data analysis pipeline that we propose here will also help to standardize variant calling in small RNA genomes based on next-generation sequencing data.

## Background

Viruses outnumber all other known life forms on earth. Furthermore, viruses in general and RNA viruses in particular have a huge genetic diversity, which is the driving force of their evolutionary success. Viral genomic diversity is well captured in the term ‘quasispecies’. The term ‘quasispecies theory’ was first introduced by Manfred Eigen as a theoretical model to study molecular evolution by mutation and selection in self-reproducing macromolecules [1,2]. Later, the term was also used to describe an RNA virus population consisting of a mixture of related genomes [3-6]. A viral quasispecies is defined as a proliferating population of non-identical but closely related viral genomes in a mutation-prone environment subjected to continuous competition and selection [5,7]. Biologically, the quasispecies is the level at which selection takes place [8]. Human influenza viruses represent a prototypical example of rapid virus evolution facilitated by error-prone genome replication combined with the selection pressure imposed by host immune responses. This situation favors the emergence of fit mutant viruses that escape the herd immunity induced by infection with parental viruses or by vaccination [9,10].

Influenza is an acute and highly contagious viral disease of the respiratory tract in humans. It is caused by influenza A and B viruses and occasionally by influenza C virus. These viruses represent three of the five genera of the *Orthomyxoviridae* family, which is characterized by enveloped viruses that have a segmented, single-stranded, negative sense RNA genome [11]. Replication of the RNA genome of influenza viruses is associated with a relatively high mutation rate (2.3 × 10^−5^) because the viral RNA-dependent RNA polymerase lacks 3′-5′-exonuclease activity and therefore has no proof-reading function [12,13]. Mutations that are introduced during replication are tolerated because they are neutral for virus fitness in a particular environment, rapidly lost because they reduce fitness, or expanded because they are advantageous [5].

The mutation rate of influenza A viruses has been traditionally determined by sequencing different cDNA clones obtained from multiple plaques descending from a plaque-purified influenza A virus [14]. In other words, viral genomes that are fit enough to generate plaques were sequenced. This approach revealed a mutation rate of approximately 1.5 × 10^−5^ per nucleotide per infectious cycle. Sequence analysis of multiple clones of cDNA fragments derived from one or more gene segments has also been used to study sequence variation of influenza virus derived from clinical samples [15,16]. In addition, deep amplicon sequencing of one or two gene segments from avian H7N1 and equine H3N8 influenza viruses has been applied to study within and between host genetic variation [17,18]. However, identification of the extent of genetic variation in a viral quasispecies under a given condition requires a highly accurate sequencing method that does not rely on molecular cloning, or a phenotypic selection method such as plaque generation. Next-generation sequencing (NGS) seems to fulfill this requirement [19-21]. However, experimental errors are introduced during the preparatory steps, *i.e.* reverse transcription and PCR amplification, and the NGS method itself is also an error-prone process [22].

NGS enables sequencing of multiple gigabases of DNA in a single run; the output size depends on the sequencing instrument [23]. Consequently, because the influenza genome consists of only 13,000 ribonucleotides, it is straightforward to sequence it at high coverage (*i.e.* the number of times the genome is sequenced). However, its segmented RNA genome makes it technically challenging to obtain full genome coverage. Stoichiometric RT-PCR amplification of each of the eight genomic RNA segments is difficult, in particular when starting from *ex vivo* samples such as nasal swabs or bronchoalveolar lavage from experimentally infected animals. NGS studies of influenza virus reported to date did not start from the amplification of all eight full-length genomic segments in sufficient amounts in a single reaction, and homogeneous coverage across all eight segments was not always obtained [24-29].

Here, we compared the suitability of two NGS methods to determine the influenza A virus quasispecies diversity. We deep-sequenced A/Puerto Rico/8/34 (PR8) influenza virus, which is used extensively in many research laboratories for *in vitro* and mouse experiments. In addition, PR8 virus is used as a donor to generate egg-grown reassortant viruses for seasonal influenza vaccine production. Importantly, we also took advantage of the available plasmid-based reverse genetics system for PR8 virus because it is a genetically stable equivalent of the virus [30]. We compared the quality of the primary sequence data, the read length, the coverage across the viral genome, the method-associated error rate, and the sensitivity of two modern NGS platforms: the Illumina MiSeq sequencing-by-synthesis and the Ion Torrent PGM semiconductor sequencing technique. For both sequencing platforms, we used the latest available software and the most recent chemistries available.

## Results

### High-throughput sequencing of plasmid samples

Our aim was twofold: (1) to compare the performance of two high-throughput sequencing instruments; (2) to determine the complexity of an influenza A virus quasispecies (to count the number of nucleotide variants present in a swarm of genomes of that virus). We selected the Illumina MiSeq and the Ion Torrent PGM sequencing platforms because the accuracy of single nucleotide polymorphism (SNP) identification of these two popular NGS platforms is unclear. A study by Quail and colleagues concluded that the overall SNP calling rate is slightly higher for the data generated by Ion Torrent PGM than for Illumina MiSeq data [21], whereas Loman and colleagues reported a lower substitution error rate for the Illumina MiSeq [20].

We first compared the accuracy and sensitivity of these two sequencers. We used plasmid DNA to compare the accuracy of the sequencing output because it is genetically very stable. We also generated a plasmid with two tracer mutations, which allowed us to prepare mixtures with different, defined amounts of wild type and mutant plasmid before sequence analysis, in order to determine the sensitivity of the sequencers for picking out the occurrence of the introduced SNPs. For this comparison, we chose plasmids that also allowed us to generate PR8 virus with or without the introduced tracer mutations [30,31].

**Figure 1:**
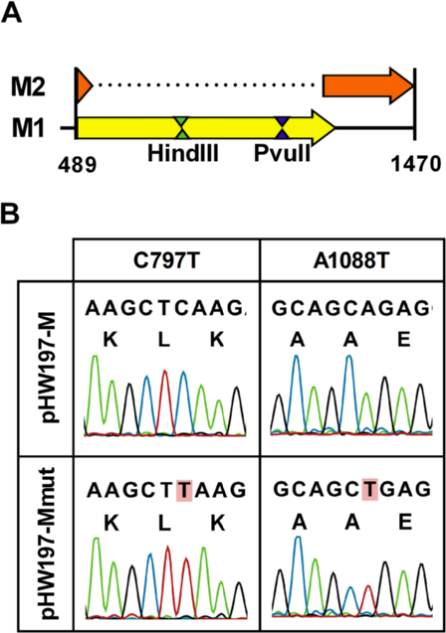
**Introduction of synonymous tracer mutations in gene segment 7 of PR8 virus. (A)** Schematic representation of the influenza M segment present in pHW197-Mmut. The open reading frames of M1 (yellow, starting at position 489, relative to the upstream CMV promoter (not depicted)) and M2 (orange, starting at position 489 and ending at position 1470) are indicated. The resulting HindIII and PvuII restriction sites are indicated. **(B)** Fluorograms showing the synonymous substitutions in pHW197-Mmut relative to pHW197-M at positions 797 (C to T) and 1088 (A to T). The predicted amino acid sequence is shown underneath the nucleotide sequence.

We generated a mutated version of plasmid pHW197-M (pHW197-Mmut). This mutant has two silent mutations in the influenza virus M1 open reading frame (ORF) that served as tracers when mixing pHW197-Mmut and pHW197-M plasmids at different ratios. Because we intended to perform such mixing experiments with both plasmids and viruses generated from these plasmids, we carefully selected two silent mutations that most likely would not affect virus fitness. We chose these mutations based on their prevalence in human H1N1 virus isolates (see Methods). We selected two silent mutations in M1, which at the same time also added a restriction site to facilitate screening (Figure 1A). These mutations were introduced in pHW197-M at positions 797 (C797T, pHW197-M numbering; C354T, segment 7 numbering) and 1088 (A1088T, pHW197-M numbering; A645T, segment 7 numbering). So the resulting plasmid, pHW197-Mmut, had additional HindIII and PvuII restriction sites. The presence of these mutations was verified by restriction analysis and conventional Sanger sequencing (Figure 1B).

#### Sequence read length

**Figure 2:**
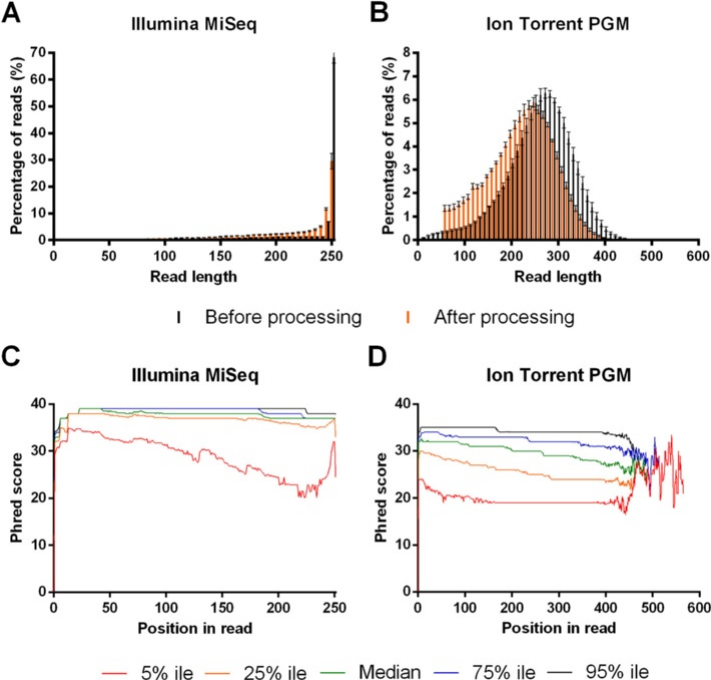
**Quality of sequencing reads obtained on the Illumina MiSeq and Ion Torrent PGM platforms.** The pHW197-M and pHW197-Mmut plasmids (= 7) were fragmented with the Nextera XT DNA sample preparation kit (Illumina MiSeq) or with Covaris mechanical shearing followed by adaptor ligation (Ion Torrent PGM). Distribution of the read lengths obtained on the Illumina MiSeq **(A)** and Ion Torrent PGM **(B)** before processing (in black, output files of sequencer) and after processing (in orange) the obtained sequencing reads. Processing implies removal of adaptor contamination, quality trimming (> Q20), the removal of ambiguous bases and removal of reads shorter than 50 bases. For the Illumina MiSeq reads, broken pairs after read processing were also removed during the processing. Error bars represent the standard deviation. **(C, D)** Per-base quality distribution of sequencing reads. The Phred score distribution (Y-axis) relative to the processed reads obtained after sequencing on the Illumina MiSeq **(C)** and Ion Torrent PGM **(D)**. x% ile = x^th^ percentile of quality scores observed at that position.

Assuming an equal error rate per base, longer read lengths are preferred for the *de novo* sequence assembly. In addition, longer read lengths increase the likelihood that one can conclude whether mutations observed in a genomic segment are linked or not. The two point mutations that we introduced in the M gene segment are 291 nucleotides apart. Therefore, to confirm the presence of these two mutations on the same DNA molecule, read lengths after processing should be at least 291 nucleotides long. Such a length should be obtained when using the Ion Torrent PGM 400 base-pair sequencing kits. The length distribution of the sequencing reads of the plasmid samples generated by both sequencers is shown in black in Figure 2. Plasmid samples were fragmented with Nextera XT transposase for Illumina MiSeq and mechanically sheared by Covaris, followed by adaptor ligation before Ion Torrent PGM sequencing. Nearly 70% of the unprocessed reads obtained on the Illumina MiSeq (2x250 bp sequencing) have a length of 250 bp, and the mean read length is 233.70 bp ± 1.65 bp (Figure 2A). The length of the unprocessed reads generated by the Ion Torrent PGM (400-bp sequencing on Ion 318 chip v2) follows a Gaussian distribution with a peak around 280 bp and a mean read length of 261.06 bp ± 2.51 bp (Figure 2B). These values are lower than expected since the Ion PGM Template OT2 400 Kit, Ion PGM Sequencing 400 Kit and Ion 318 chip v2 (revision 2.0) that we used should offer sequence reads of 400 bp according to their manuals. As analyzed on a High Sensitivity DNA Chip on the Agilent Bioanalyzer, the peak fragment size before emulsion PCR (emPCR) was situated around 450 bp (data not shown), indicating that Covaris shearing and subsequent size selection did not account for this relatively short average sequence length. We note that Junemann et al. also obtained fragments with the OT2 400 kit that were shorter than expected [19].

#### In silico processing of the sequencing reads

**Figure 3:**
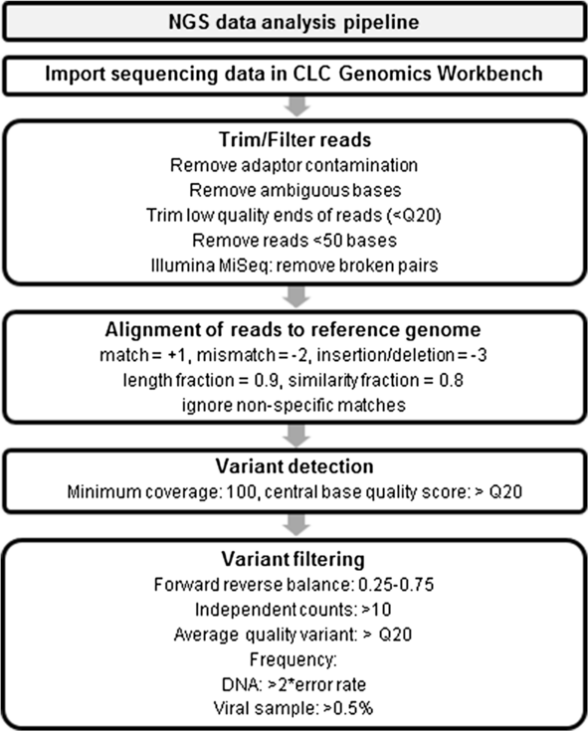
**Next generation sequencing data analysis pipeline.** Schematic representation of the analysis pipeline for *in silico* processing of next-generation sequencing data.

Accurate analysis of viral quasispecies composition has to be based on high quality reads to ensure that SNPs and insertions and deletions (indels) can be confidently counted, because low quality reads could lead to over-interpretation of the number of mutations. In addition, high quality reads will lead to a higher accuracy of *de novo* sequence assembly. Therefore, we performed a quality control using the CLC Genomics Workbench software; we also propose a NGS data analysis pipeline that is generally applicable (Figure 3). First, we removed adaptor contamination and the low quality ends of the sequencing reads from the data generated by the two deep sequencing techniques. It was recently reported that applying a Phred score of 20 or higher to filter Illumina MiSeq NGS data dramatically reduces the noise in SNP calling [32]. Hence, we applied this quality threshold to all our plasmid-derived sequencing reads. A Phred score is logarithmically related to the base-calling error probabilities. When a Phred score of 20 is assigned to a base, it means that the chance that this base is called incorrectly is 1 in 100. We also discarded ambiguous bases and read lengths below 50 bases, which further reduces the background because such short reads are often mapped inaccurately. This quality trimming and read length filtering retained 94.89% ± 0.55% of the Illumina MiSeq and 95.26% ± 0.57% of the Ion Torrent PGM reads. On the other hand, 85.99% ± 0.72% of the bases sequenced on the Illumina MiSeq and 78.99% ± 1.22% of the bases sequenced on the Ion Torrent PGM were retained. This indicates that the greatest loss of bases was due to quality trimming rather than read length filtering and that Illumina MiSeq sequencing provides higher sequencing quality than Ion Torrent PGM. The resulting read length distribution after this *in silico* filtering is shown in orange in Figure 2, where the mean read length is 211.78 bp ± 2.18 bp on the Illumina MiSeq and 216.43 bp ± 1.15 bp on the Ion Torrent PGM after processing of the reads.

#### Quality of the sequencing reads

The per-base quality distribution on both sequencers, using the plasmid samples as template, is shown in Figure 2. Bases with a Phred score of 30 (chance of a wrong base call of 1 in 1000) are a measure of high quality data. For the processed reads obtained on the Illumina MiSeq, the 25th percentile of the Phred scores is ≥ 33 until position 251, and thus most of the sequencing reads are without sequencing error (Figure 2C). For the reads obtained on the Ion Torrent PGM, the median of the Phred scores is ≥ 30 until position 266 (Figure 2D). Therefore, we conclude that the overall sequencing quality of the reads obtained on the Illumina MiSeq is higher than that obtained on the Ion Torrent PGM.

#### Mapping of the sequencing reads

To evaluate the accuracies of both sequencers, the processed reads were mapped to the plasmid reference sequence (Table 1). The percentage of unmapped bases was lower for the Illumina MiSeq (0.17% ± 0.02%) than for the Ion Torrent PGM (1.14% ± 0.10%). This is due to the lower quality of the Ion Torrent PGM sequencing reads, which reflects the intrinsic sequencing errors that lead to reduced alignment and a higher number of unmapped bases, particularly at the ends of the longer reads.

**Table 1:** **Alignment metrics for Illumina MiSeq and Ion Torrent PGM sequencing runs**

	**Illumina MiSeq**				**Ion Torrent PGM**			
	**pHW197-M**		**pHW197-Mmut**		**pHW197-M**		**pHW197-Mmut**	
	**S1**	**S2**	**S1**	**S2**	**S1**	**S2**	**S1**	**S2**
**Minimum coverage**	683	744	815	609	3532	4510	3995	4830
**Maximum coverage**	27389	28589	32802	26275	15716	17632	14664	18196
**Average coverage**	15369	16315	18236	14610	11525	13236	11118	13636
**Standard deviation**	6739	7120	7888	6315	3323	3499	2853	3562
**Unmapped reads (%)**	0.20	0.16	0.21	0.22	1.06	1.05	1.28	1.19
**Unmapped bases (%)**	0.17	0.14	0.19	0.19	1.07	1.05	1.26	1.18

Wild type (pHW197-M) and mutant (pHW197-Mmut) plasmids were sequenced in duplicate (S1 and S2) on both sequencers and the processed reads were mapped to the plasmid reference sequence.

**Figure 4:**
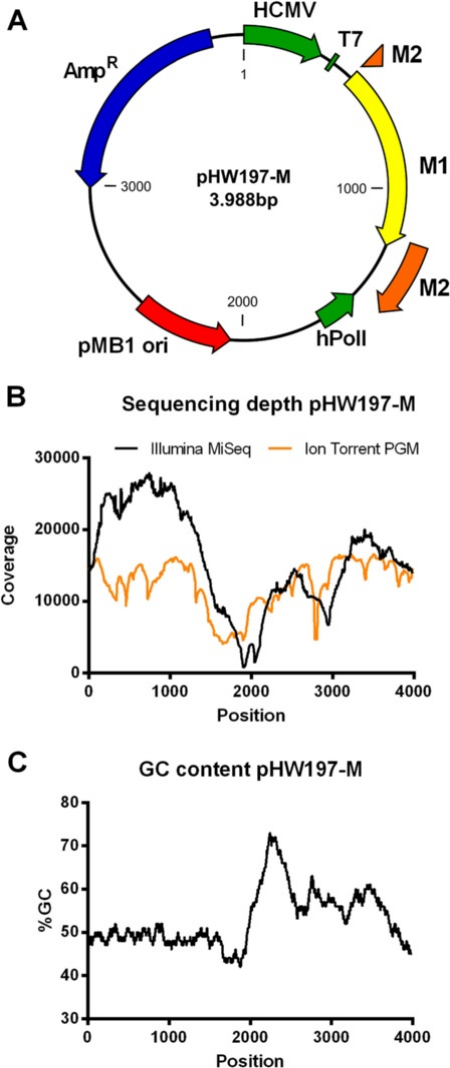
**Next generation sequence analysis of pHW197-M. (A)** Schematic representation of pHW197-M. HCMV: human cytomegalovirus promoter, T7: T7 RNA polymerase promoter, M1: matrix protein 1 open reading frame, M2: matrix protein 2 open reading frame (interrupted by an intron), hPolI: human RNA polymerase I promoter, pMB1 ori: origin of replication, Amp^R^: ampicillin resistance gene. **(B)** Mean sequencing depth after mapping the processed reads (n = 2) to the reference plasmid genome. The pHW197-M plasmid was fragmented with the Nextera XT DNA sample preparation kit before Illumina MiSeq sequence analysis or by Covaris mechanical shearing, followed by adaptor ligation before Ion Torrent PGM sequence analysis. **(C)** Percentage GC distribution in the pHW197-M plasmid reference sequence. The peak after position 2000 corresponds to the origin of replication.

For both sequencers, we observed a striking fluctuation in coverage depth (times a nucleotide is sequenced plotted against the position in the genome) (Figure 4). The largest fluctuation was seen for the Illumina MiSeq (Figure 4B). It is known that Illumina MiSeq and Ion Torrent PGM sequencers perform rather poorly when sequencing DNA with very low or very high GC content, which leads to low sequencing coverage of AT and GC rich regions [33,34]. In addition, the Nextera transposon-based fragmentation that we used for the samples sequenced on the Illumina MiSeq has some sequence preference, which can lead to a fragmentation bias, particularly in small genomes [35].

**Figure 5:**
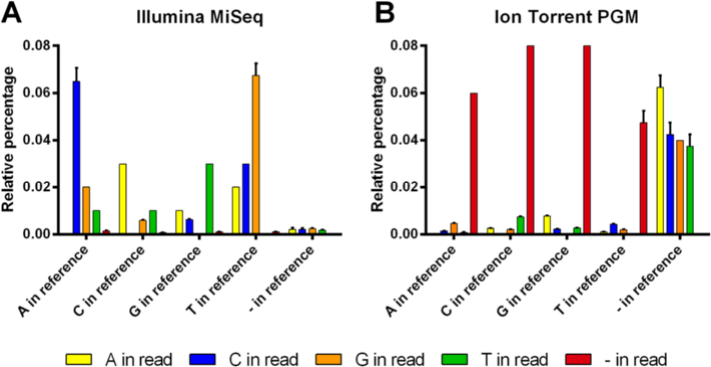
**Comparison of nucleotide variants revealed by Illumina MiSeq and Ion torrent PGM sequencing.** The pHW197-M and pHW197-Mmut plasmids were fragmented with the Nextera XT DNA sample preparation kit (Illumina MiSeq) or by Covaris mechanical shearing, followed by adaptor ligation (Ion Torrent PGM). The samples were sequenced in duplicate and the sequence reads were processed (adaptor removal, Q20 trimming, removal of ambiguous bases and removal of reads shorter than 50 bases). For reads obtained on the Illumina MiSeq: broken pairs after read processing were also removed. The relative percentages of substitutions, insertions and deletions were determined after mapping the processed Illumina MiSeq **(A)** and Ion Torrent PGM **(B)** sequencing reads to the pHW197-M (n = 2) or pHW197-Mmut (n = 2) reference sequence. Bars represent averages from 4 samples and error bars represent the standard deviation.

Since the plasmid reference sequence is known, we were confident that any mismatching nucleotide variant could be reported as a sequencing error. The error rate per read position was 0.08% ± 0.01% for the Illumina MiSeq and 0.12% ± 0.01% for the Ion Torrent PGM. The error rate increases slightly with the read length for both sequencers, with a pronounced rise at the end of the reads on the Ion Torrent PGM (data not shown). For the Illumina MiSeq, substitutions are the dominant error type with A-to-C and T-to-G being the most prevalent (Figure 5A), which is consistent with an earlier report [36]. In contrast, indels are dominant on the Ion Torrent PGM (Figure 5B), and most of them are single nucleotide insertions or deletions (data not shown). Nearly all of these indels occur in homopolymeric regions. Since these regions require multiple incorporations of identical nucleotides, this increases the chance of non-linearity between the signal intensity and homopolymer length, explaining the higher indel error rate of the Ion Torrent PGM.

#### Variant detection

We considered the frequency of a given nucleotide significant (a real mutation) when it was higher than twice the sequencing error background, *i.e.* above 0.16% for the Illumina MiSeq and above 0.24% for the Ion Torrent PGM. Since we are dealing with proportions very close to zero, the proportion of variants that could be miscalled at this threshold was estimated using the Agresti-Coull interval as an approximate binomial confidence interval [37]. Setting twice the background error rate as upper bound of the binomial confidence interval, only 0.0041% and 0.00002% of the variants are expected to be miscalled as true variant on the Illumina MiSeq and Ion Torrent PGM, respectively. Despite this stringent cut-off, false positive errors were still detected, mostly as a consequence of the sequence specific error profiles of both sequencers (Table 2, [21,38,39]). The largest number of variants was deduced from the Ion Torrent PGM data, and all of them were indels (Table 2). In contrast, the variant calls on the Illumina MiSeq were mainly SNPs (Table 2). To eliminate false positive variants, we applied extra *in silico* filtering parameters. We set the forward/reverse balance between 0.25 and 0.75, meaning that the minimum ratio between the number of forward and reverse reads that support the surmised variant should be at least 0.25. In addition, a nucleotide variant should be counted at least 10 times independently and should have an average Phred score of at least 20 (based on [40]) (Figure 3). Applying these variant filters removed most of the false positive variant calls and retained one variant from the Illumina MiSeq and six or five variants from the Ion Torrent PGM data (Table 2). So applying the variant filtering parameters has the largest impact on removing false positive variants detected in the Ion Torrent PGM data. Regardless of the sequencing method used, all false positive indels were present in homopolymer regions (at least two consecutive identical bases in the plasmid reference sequence). These variants can be excluded by using a homopolymer indel filter. However, homopolymeric regions are also the sites were the viral RNA polymerase may have the highest error rate. Therefore, applying this homopolymer indel filter to analyze viral RNA sequences (see below) could lead to underestimation of the number of variant genomes. Alternatively, the number of called variants based on the Ion Torrent PGM data can be reduced in order to exclude likely false positive variants, by increasing the average Phred score for a registered variant to 30. However, this also increased the number of false negative variant calls (data not shown).

**Table 2:** **Number of detected variants in the pHW197-M sample before and after filtering**

	**Illumina MiSeq**				**Ion Torrent PGM**			
	**Before**		**After** ^**a**^		**Before**		**After** ^**a**^	
	**S1** ^**b**^	**S2** ^**b**^	**S1** ^**b**^	**S2** ^**b**^	**S1** ^**b**^	**S2** ^**b**^	**S1** ^**b**^	**S2** ^**b**^
**SNP** ^**c**^	4	4	0	0	0	0	0	0
**Insertion**	0	0	0	0	14	12	3	1
**Deletion**	0	2	0	1	71	66	3	4

^a^The filtering parameters used were average quality threshold > Q20, forward/reverse balance > 0.25, and independent counts of variant > 10.

^b^Sequencing was performed in duplicate (S1 and S2).

^c^SNP = single nucleotide polymorphism.

To determine the sensitivity for variant calling, we mixed pHW197-M and pHW197-Mmut plasmids in ratios of 95:5, 99:1 and 99.9:0.1 (v:v) and then sequenced the mixtures on both platforms. On both sequencers, the calculated frequency of pHW197-M or pHW197-Mmut based on the output data closely resembled the used ratios (Table 3). Nevertheless, the average quality (average Phred score) of the tracer mutations was higher on the Illumina MiSeq (37.97 ± 0.09) than on the Ion Torrent PGM (30.72 ± 1.07), making the detected variants on the Illumina MiSeq more reliable. Since the mutations are physically linked on one plasmid, both mutations should be present at similar frequencies in a single sample. This was indeed the case: the observed frequencies of the linked tracer mutations varied only slightly with on average 0.18% ± 0.26% on the mapped Illumina MiSeq reads and 0.22% ± 0.15% on the mapped Ion Torrent PGM reads. Finally, we found that the 99.9:0.1 plasmid input ratio could not be resolved because it is too close to the intrinsic error rate of both sequencers. Overall, the Illumina MiSeq is more accurate than the Ion Torrent PGM sequencer but they have similar sensitivities for detection of SNPs in plasmid DNA.

**Table 3:** **Sensitivity of Illumina MiSeq and Ion Torrent PGM**

		**Illumina MiSeq**				**Ion Torrent PGM**			
		**797**		**1088**		**797**		**1088**	
**pHW197-M**	**pHW197-Mmut**	**C**	**T**	**A**	**T**	**C**	**T**	**A**	**T**
0	100	< d.l.	99.97	< d.l.	99.96	< d.l.	99.56	< d.l.	99.89
0	100	< d.l.	99.95	< d.l.	99.94	< d.l.	99.62	< d.l.	99.96
95	5	94.84	5.14	95.40	4.58	95.22	4.75	95.02	4.96
99	1	98.78	1.19	98.93	1.06	98.97	1.02	98.96	1.02
99.9	0.1	99.80	0.17	99.85	< d.l.	99.93	< d.l.	99.87	< d.l.

The observed mutation frequencies (%) after mapping the reads to the reference sequence of pHW197-M are shown. < d.l. = mutation frequency falls below detection limit (< 2*error rate, < 0.16% for Illumina MiSeq and < 0.24% for Ion Torrent PGM). The pHW197-Mmut plasmid contains the tracer mutations C797T and A1088T.

### Sequencing of influenza virus samples

**Figure 6:**
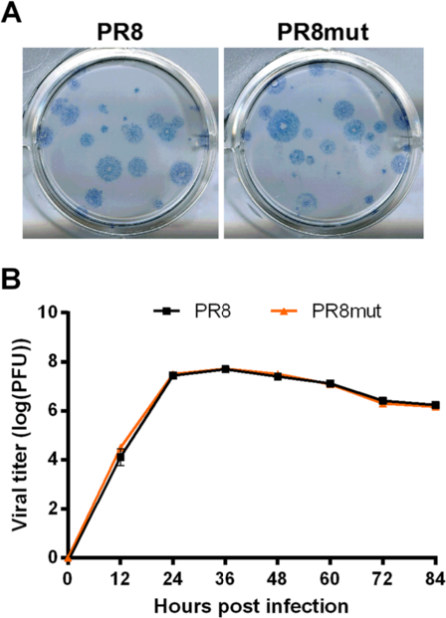
**Comparison of the** ***in vitro*** **replication of PR8 and PR8mut influenza viruses. (A)** Individual plaques of PR8 and PR8mut. Plaques were revealed by immunostaining with an anti-M2 ectodomain-specific monoclonal antibody. **(B)** Multi-cycle growth analysis of PR8 and PR8mut viruses. MDCK cells were infected in triplicate at a MOI of 0.01 of PR8 or PR8mut virus. Every twelve hours after infection, samples in the cell supernatant were analyzed for the presence of infectious virus by plaque assay. Error bars represent the standard deviation.

To compare the efficacy of the sequencers for detecting mutations in an influenza A virus sample, we generated influenza virus starting from eight plasmids, including pHW197-M or pHW197-Mmut. This resulted in wild type PR8 and mutant PR8 (PR8mut), respectively, the latter carrying two silent mutations in the M1 ORF (C354T and A645T, segment 7 numbering). These mutations did not seem to affect viral fitness because PR8 and PR8mut replicated equally well *in vitro* (Figure 6). In addition, Sanger sequencing and restriction analysis of the mutant M segment after RT-PCR revealed that the introduced tracer mutations in PR8mut were uniformly present in the stock preparation (data not shown). These viral samples were sequenced in duplicate (*i.e.* from each RT-PCR sample two libraries of DNA fragments were generated in parallel) to evaluate the consistency of the two NGS methods. In addition, wild type and mutant viruses were mixed at a ratio of 99:1 before RNA isolation to compare the accuracy of the two NGS sequencing methods to resolve this ratio. Finally, we also wanted to quantify the number of differences, if any, between the plasmid encoded influenza virus information and the *in vitro* cultured virus samples. This quantification would reflect the baseline quasispecies diversity, in the absence of exogenous selection pressure.

#### Amplification of the genomic influenza virus segments

**Figure 7:**
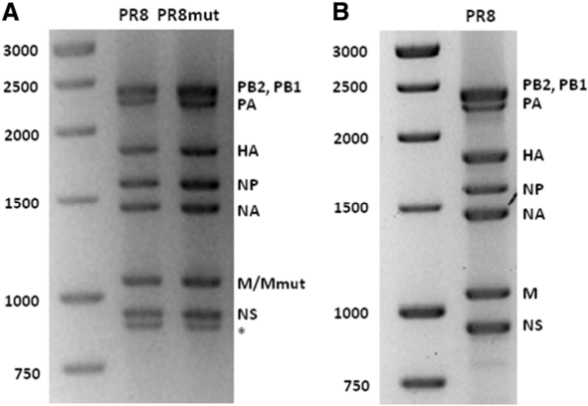
**RT-PCR amplification of influenza A virus PR8 and PR8mut genomic RNA. (A)** Electrophoretic analysis of RT-PCR products of PR8 and PR8mut separated on a 1.5% agarose gel and subsequently stained with Ethidium Bromide. PB1: polymerase basic 1, PB2: polymerase basic 2, PA: polymerase acidic, HA: hemagglutinin, NP: nucleoprotein, NA: neuraminidase, M: matrix, NS: non-structural. The amplified PB1 and PB2 RT-PCR products run at the same position in the gel. * = aspecific amplification product of 847 bp. **(B)** Optimized RT-PCR product resolved as in A.

Ensuring sufficient coverage across all segments requires an RT-PCR protocol that amplifies all eight influenza genome segments with equal efficiency. We used an RT-PCR protocol based on the conserved termini of the influenza genome segments, which allowed us to amplify all eight segments in sufficient amounts (Figure 7A) [41-43]. Surprisingly, next to the eight genomic segments, an unexpected band with a length of about 850 bp was also amplified. This band was identified by conventional Sanger sequencing after blunt-end cloning in pBlueScript and corresponded to the first 847 nucleotides of HA. Its amplification in the RT-PCR reaction was probably due to partial overlap of the CommonUni12G primer with a nine-nucleotide perfect match in the coding region of HA (GCCGGAGCTCTGCAGATATCA**GCGAAAGCA**GG, match in bold). By lowering the concentration of the CommonUni12G primer, we could avoid this extra band and obtained the eight amplicons of the expected size (Figure 7B). Overall, these results show that this RT-PCR protocol based on the conserved termini of the influenza A genome segments is suitable for amplifying all eight segments simultaneously and efficiently.

#### De novo assembly of sequencing reads derived from viral RNA

Accurate *de novo* nucleotide sequence assembly is essential to identify the viral quasispecies that is present in (clinical) samples. The viral RT-PCR products were purified and subjected to NGS on the Illumina MiSeq and the Ion Torrent PGM platforms. Before assembly, the reads were processed *in silico* as described above for the plasmid-derived sequences (Figure 3). Afterwards, the sequencing reads were assembled *de novo* using de Bruijn graphs [44]. This assembly method is ideally suited for high coverage next-generation sequencing data since the computational burden is lowered by first subdividing all sequencing reads in all possible subsequences with a certain short length (k), followed by looking for all neighbors with k-1 overlap. The consensus sequence is then constructed as being the alignment of k-mers that follows the shortest path connecting all overlap sequences [45]. In this way, 99.90% ± 0.02% of the reads on the Illumina MiSeq and 99.65% ± 0.16% of the reads on the Ion Torrent PGM were assembled in eight contigs corresponding to the eight genome segments of the PR8 virus. These eight contigs had a mean coverage depth of 23020 ± 3504 on the Illumina MiSeq and 13768 ± 394 on the Ion Torrent PGM. All viral genome segments were almost completely covered by the consensus contigs (Table 4). Only the extreme 3′ and 5′ ends of each segment were not covered in all consensus sequences. This is partly due to the high sequence similarity and partial complementarity of the 5′ and 3′ ends of the influenza virus genome, making those reads more difficult to assemble *de novo*. In addition, the transposase-based fragmentation and tagging of the samples sequenced on the Illumina MiSeq disfavors coverage of free ends, making *de novo* assembly at these ends more difficult. For the Ion Torrent PGM samples, the adaptors were ligated to the DNA fragments that had been generated by sonication, with the free ends of the influenza genome DNA segments favoring adaptor ligation, resulting in higher coverage of the segment termini and making full-length *de novo* assembly easier. Nevertheless, in all viral contigs, the coding sequences were highly covered and entirely present. In summary, both sequencers are equally suited for *de novo* assembly of the influenza virus genome, and transposase-based fragmentation should be avoided when high coverage of the influenza virus genome ends is desired.

**Table 4:** **Percent coverage of the influenza PR8 reference sequence after** ***de novo*** **assembly**

**Segment**	**Illumina MiSeq** ^**a**^ **(SD** ^**b**^ **)**	**Ion torrent PGM** ^**a**^ **(SD** ^**b**^ **)**
**PB2**	99.55 (0.30)	100.00 (0.00)
**PB1**	99.37 (0.52)	100.00 (0.00)
**PA**	99.35 (0.54)	99.30 (0.21)
**HA**	98.65 (0.52)	99.04 (0.50)
**NP**	98.79 (0.92)	98.07 (0.00)
**NA**	98.92 (0.82)	99.97 (0.07)
**M - Mmut**	98.20 (1.36)	99.55 (0.89)
**NS**	96.94 (0.76)	98.17 (2.12)

^a^Viral RT-PCR product sequencing reads obtained on Illumina MiSeq and Ion Torrent PGM were *de novo* assembled, followed by alignment of the obtained consensus sequence to the PR8 (n = 2) or PR8mut (n = 2) reference genome. For each segment, the percentage of the influenza reference sequence (based on the sequence from the plasmids from which the virus was produced) that is covered by the assembled contigs is given.

^b^SD = standard deviation.

#### Mapping of sequencing reads

**Figure 8:**
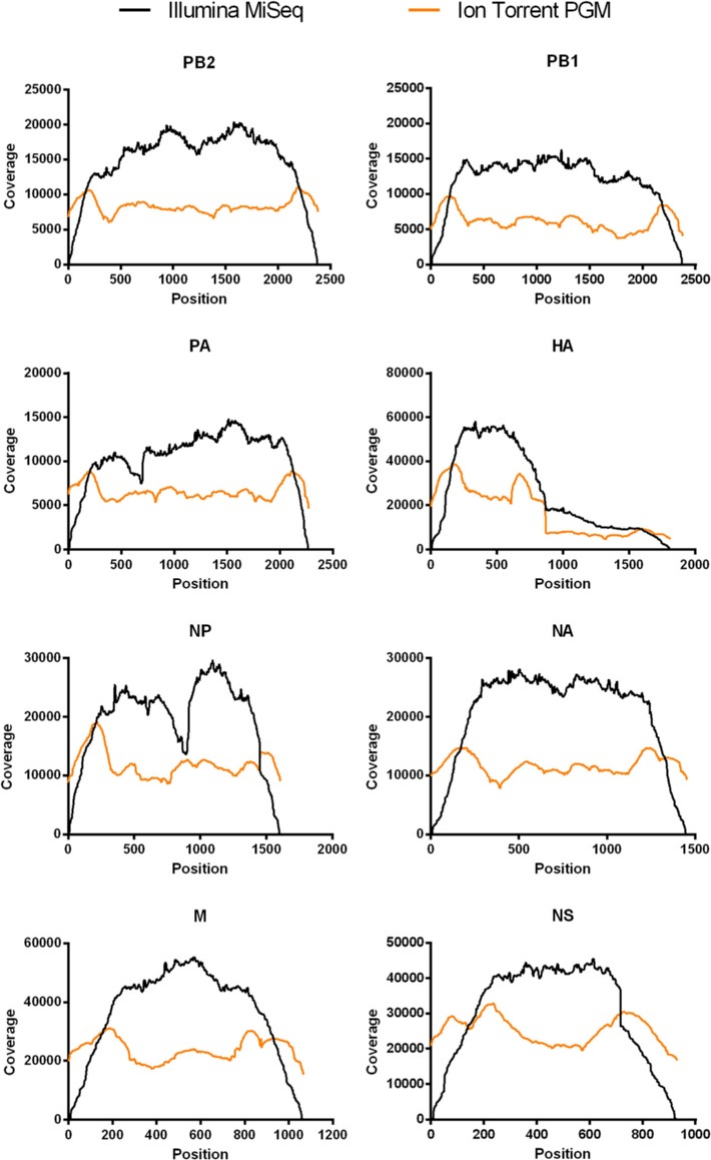
**Sequence coverage of the influenza virus genome.** Sequence coverage for the different genome segments of wild type PR8 virus sequenced on Illumina MiSeq (2x250 bp, black lines, n = 2) or Ion Torrent PGM (Ion 318 chip v2, orange lines, n = 2). The obtained sequences were mapped to the reference genome (based on the pHW plasmids that were used to generate the virus, with addition of the extra 20 nucleotides present at the 5′ site in the RT-PCR primers).

**Figure 9:**
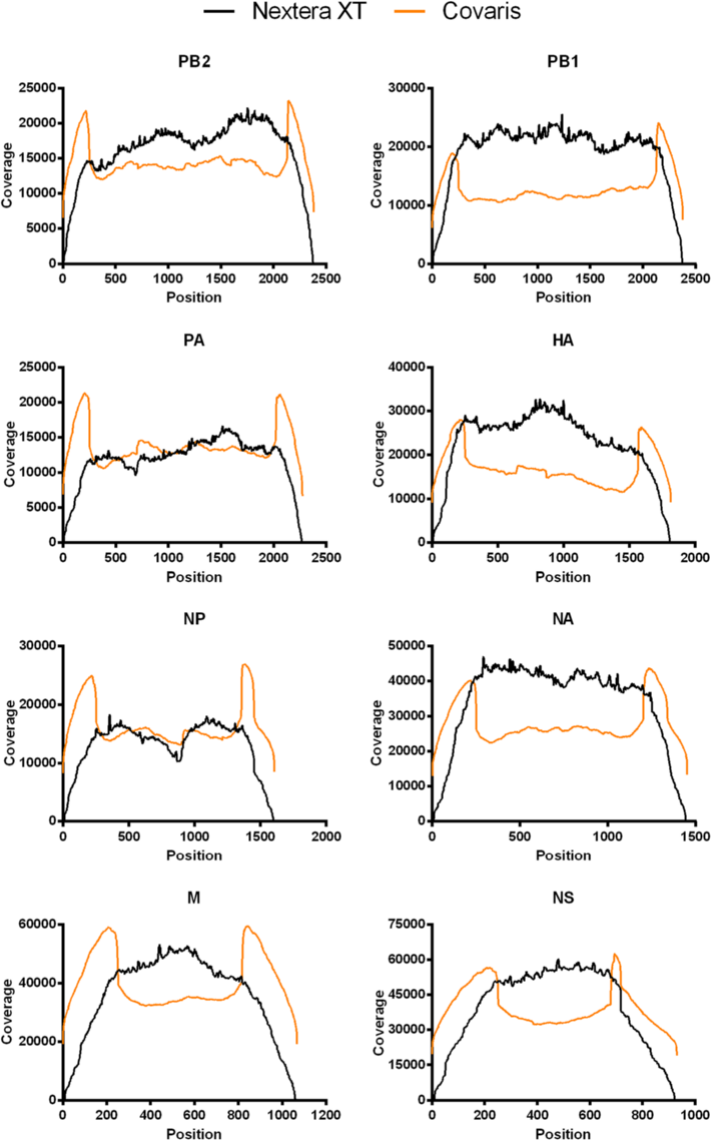
**Coverage of PR8 virus genome with the optimized RT-PCR protocol.** Sequence coverage for the different genome segments of wild type PR8 virus sequenced on Illumina MiSeq (2x250 bp) using two different fragmentation methods: Nextera XT transposase-based fragmentation (black lines) and mechanical Covaris shearing followed by adaptor ligation (orange lines). The obtained sequences were mapped to the reference genome (based on the plasmids used to generate the virus).

Mapping of the above-mentioned reads to the viral reference genome (based on the eight plasmids used to generate the recombinant PR8 virus, with addition of the extra 20 nucleotides present at the 5′ site in the RT-PCR primers) resulted in sufficient full-length coverage of the entire influenza genome (Figure 8 and Table 5). This allowed us to study the viral quasispecies, *i.e.* to determine the number of variable nucleotides at each position in the viral genome. When mapping was done with the Illumina MiSeq data, we noticed a significant coverage dip near the middle of the NP segment as well as a dip around position 600 of the PA segment, but this did not occur when the Ion Torrent PGM data were used (Figure 8). These parts of NP and PA are not particularly GC-rich or AT-rich, and these coverage dips therefore likely reflect a sequence dependency of the Nextera transposase [35,46]. Indeed, when we used mechanical shearing to fragment the RT-PCR products before Illumina MiSeq sequencing, coverage of the NP and PA segments was high and consistent over the entire length of all PR8 genome segments (Figure 9, orange). For the viral samples sequenced on the Ion Torrent PGM, the sequencing depth is more homogenous across the segments, and the regions close to the ends of the viral segments are slightly overrepresented. This overrepresentation is probably due to mechanical shearing and subsequent adaptor ligation. The inadvertent RT-PCR amplification of the 847-bp HA fragment mentioned earlier was clearly reflected in the sequence read coverage of that segment, which showed a higher coverage for the 5′ half of this segment (Figure 8). Moreover, the gradual *versus* steep drop of coverage near position 847 in the HA segment reflects the different chemistries of the Nextera transposase and the Covaris shearing/adapter ligation methods. Homogenous coverage across the HA segment was evident with the optimized RT-PCR method in which the extra partial HA-fragment was not present (Figure 9).

**Table 5:** **Alignment metrics for Illumina MiSeq and Ion Torrent PGM sequencing runs**

**Illumina MiSeq**					
**PR8 S1**					
**Segment**	**Length**	**Mapped reads**	**Minimum coverage**	**Maximum coverage**	**Average coverage**
**PB2**	2381	159869	12	20525	15057
**PB1**	2381	126244	6	16513	11960
**PA**	2273	107490	7	14883	10533
**HA**	1815	213169	6	58709	25756
**NP**	1605	149599	9	29927	19883
**NA**	1453	139858	5	29353	21256
**M**	1067	180592	13	56656	37788
**NS**	930	140785	4	47293	31651
**PR8 S2**					
**Segment**	**Length**	**Mapped reads**	**Minimum coverage**	**Maximum coverage**	**Average coverage**
**PB2**	2381	163969	14	20266	14923
**PB1**	2381	128791	9	16043	11750
**PA**	2273	110954	5	14733	10486
**HA**	1815	222513	5	57511	25860
**NP**	1605	150831	11	29497	19330
**NA**	1453	135597	14	27006	19834
**M**	1067	177520	13	54233	35854
**NS**	930	136505	12	44068	29591
**Ion torrent PGM**					
**PR8 S1**					
**Segment**	**Length**	**Mapped reads**	**Minimum coverage**	**Maximum coverage**	**Average coverage**
**PB2**	2381	93676	6396	11399	8765
**PB1**	2381	72187	4016	10132	6471
**PA**	2273	70492	4735	9315	6940
**HA**	1815	148242	4613	39585	17544
**NP**	1605	94509	8518	19617	12324
**NA**	1453	77561	7918	14959	11904
**M**	1067	119301	15170	31854	24331
**NS**	930	112041	16425	33280	25428
**PR8 S2**					
**Segment**	**Length**	**Mapped reads**	**Minimum coverage**	**Maximum coverage**	**Average coverage**
**PB2**	2381	84783	5612	10775	7947
**PB1**	2381	65635	3442	9253	5900
**PA**	2273	63994	4529	8607	6290
**HA**	1815	139240	4438	37662	16517
**NP**	1605	88966	8283	18590	11625
**NA**	1453	74318	7629	14494	11453
**M**	1067	115512	15395	30397	23553
**NS**	930	109661	16936	32481	24950

Wild type PR8 virus was sequenced in duplicate (S1 and S2) on both sequencers and the processed reads were mapped to the reference sequence (based on the sequence obtained from the plasmids from which the virus was produced, with addition of the extra 20 nucleotides present at the 5′ site in the RT-PCR primers).

#### Analysis of the viral quasispecies

After mapping the reads to the reference genome, we called the variants using the optimal parameters described above (Figure 3). Since we started with viral RNA, we increased the background threshold for variant calling to 0.5%, what we believe is the biologically relevant frequency threshold. This value is above the estimated total error rate (including errors introduced by the virus itself) obtained after mapping all sequencing reads to the PR8 reference genome, which is 0.10% ± 0.01% on Illumina MiSeq and 0.12% ± 0.01% on Ion Torrent PGM.

PR8, PR8mut and a mixture of PR8 and PR8mut (99% PR8:1% PR8mut, v:v, virus samples mixed before RNA isolation), were used to prepare RT-PCR products that were subsequently sequenced on both platforms (in duplicate, except for the mixed sample) (Figure 7A). All obtained sequences were aligned to the PR8 reference genome. The output data of both sequencing platforms were processed *in silico* as described above and used to count the number of reads with C/T at position 354 and A/T at position 645 in the M segment. Illumina MiSeq slightly overestimated and Ion Torrent PGM slightly underestimated the expected percentage of tracer mutations in the PR8:PR8mut mix (Table 6). As the two introduced mutations are linked, we expected to retrieve them with the same frequency. This was indeed the case, and the observed frequencies of the linked tracer mutations differed on average by only 0.05% on the mapped Illumina MiSeq reads and by 0.27% on the mapped Ion Torrent PGM reads.

**Table 6:** **Sensitivity of Illumina MiSeq and Ion Torrent PGM to detect mutations in viral samples**

		**Illumina MiSeq**				**Ion Torrent PGM**			
		**354**		**645**		**354**		**645**	
**PR8**	**PR8mut**	**C**	**T**	**A**	**T**	**C**	**T**	**A**	**T**
0	100	< 0.5	99.96	< 0.5	99.95	< 0.5	99.61	< 0.5	99.95
0	100	< 0.5	99.96	< 0.5	99.95	< 0.5	99.62	< 0.5	99.95
99	1	98.21	1.77	98.31	1.64	98.90	0.76	98.95	0.90

The observed mutation frequencies (%) after mapping the reads of the PR8 and PR8mut viral samples to the wild type PR8 viral reference genome (based on the sequence from the plasmids from which the virus was produced) are shown. The PR8mut virus contains the tracer mutations C354T and A645T.

Next, we determined the number of variants at each nucleotide position in the virus-derived sequences, which would reflect the quasispecies diversity of *in vitro* grown PR8 and PR8mut virus. Sequencing each sample in duplicate and simultaneously on the same machine also allowed us to determine and compare the intrinsic variability of the two platforms. The number and types of nucleotide variants that were retained after applying the variant filter are presented in Table 7. Most variants were present in both sequencing duplicates, with the highest proportion of shared variants on the Illumina MiSeq (Table 7). However, the variants that were identified in only one of the duplicates were actually also detectable in the duplicate sample, but just below one of the four variant filtering parameters. As for the plasmid samples, all of the indels in the samples sequenced on the Illumina MiSeq and most of the indels in the samples sequenced on the Ion Torrent PGM were present in homopolymer regions. The frequencies of the sequencing variants detected by both sequencers in duplicate are presented in Tables 8 (PR8) and 9 (PR8mut). This revealed 19 mutations (18 SNPs and 1 deletion) for wild type PR8 and 29 SNPs for PR8mut. Nearly all SNPs were detected with a higher average Phred score on the Illumina MiSeq (37.39 ± 0.43 for PR8) and were thus more reliable than on the Ion Torrent PGM (28.58 ± 2.44 for PR8).

**Table 7:** **Number of variants detected in wild type and mutant PR8 quasispecies after filtering**

		**Illumina MiSeq**			**Ion Torrent PGM**			**Shared**
		**S1** ^**a**^	**S2** ^**a**^	**shared**	**S1** ^**a**^	**S2** ^**a**^	**Shared**	
**PR8**	**SNP** ^**b**^	25	26	24	19	21	18	18
	**Insertion**	0	0	0	1	1	0	0
	**Deletion**	6	5	4	9	9	3	1
**PR8mut**	**SNP** ^**b**^	48	46	46	32	37	32	29
	**Insertion**	0	0	0	4	4	4	0
	**Deletion**	5	6	5	8	11	4	0

The filtering parameters were: average quality threshold > Q20, forward/reverse balance > 0.25, independent counts of variant > 10, and frequency > 0.5%.

^a^The wild type and mutant PR8 quasispecies were sequenced in duplicate (S1 and S2). ^b^SNP = single nucleotide polymorphism.

**Table 8:** **Wild type PR8 quasispecies sequenced in duplicate on both Illumina MiSeq and Ion Torrent PGM**

						**Frequency (in %)**				
**Segment**	**Position**	**Type**	**Reference**	**Allele**	**aa change**	**Illumina MiSeq**		**Ion Torrent PGM**		**Function/location**
PB1	1482	Deletion	A	-	frameshift	1.87	2.19	3.18	2.88	
**PB1**	**1486**	**SNP**	**A**	**G**	**Lys481Arg**	2.32	2.62	1.91	1.91	K481 crucial for polymerase function *in vivo*, not *in vitro* [47]
PA	539	SNP	A	G	silent	1.37	1.42	0.56	0.54	/
**HA**	**607**	**SNP**	**A**	**G**	**silent**	1.60	1.56	2.02	1.85	/
HA^#^	659	SNP	G	A	Glu203Lys	1.13	1.23	0.65	0.60	enhanced receptor binding activity [48]
HA	660	SNP	A	G	Glu203Gly	3.11	3.02	1.76	1.55	slightly increased α2-6 and decreased α2-3 binding [49]
**HA**	**747**	**SNP**	**A**	**G**	**Glu232Gly**	11.56	11.43	7.29	7.19	receptor specificity [50]
HA	764	SNP	G	A	Asp238Asn	0.83	0.80	0.65	0.60	enables binding to α2.3- and α2.6-linked sialic acids [51]
**HA**	**765**	**SNP**	**A**	**G**	**Asp238Gly**	39.73	39.43	35.33	35.00	enables binding to α2.3- and α2.6-linked sialic acids [52,53]
HA	768	SNP	A	G	Gln239Arg	2.81	3.12	1.43	1.23	preferential binding to α-2,3-linked glycans [52]
**HA**	**823**	**SNP**	**A**	**G**	**Ile257Met**	1.76	1.54	0.72	0.74	located in head domain close to Sa antigenic site [54]
**HA** ^**#**^	**1199**	**SNP**	**A**	**G**	**Ser383Gly**	1.41	1.15	1.14	1.29	located in stem domain
**HA**	**1330**	**SNP**	**A**	**G**	**silent**	1.59	1.50	1.02	1.20	/
HA	1424	SNP	G	A	Val458Met	95.25	95.67	97.85	97.77	located in stem domain
HA	1440	SNP	A	G	Glu463Gly	1.91	1.75	0.58	0.56	located in stem domain
HA	1451	SNP	A	G	Ser467Gly	0.70	0.81	0.62	0.63	located in stem domain
NP	212	SNP	C	T	silent	1.80	1.76	0.83	0.64	/
NP	1249	SNP	A	G	Asn395Ser	10.71	11.01	5.97	6.76	located in NP-NP and NP-PB2 interaction domain [55,56]
NP	1324	SNP	T	G	Phe420Cys	3.43	3.41	1.31	1.15	located in the hypervariable NP_418-426_ CTL epitope [57]

^#^not present in Genbank or Influenza Research Database.

Bold = variant also present in PR8mut quasispecies.

HA segment = numbering of HA amino acid residues is based on the PR8 HA open reading frame with the starting methionine as position = 1.

**Figure 10:**
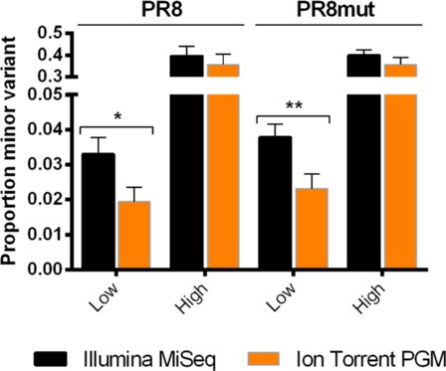
**Low frequency minor alleles are detected at significantly higher frequencies by Illumina MiSeq compared to Ion Torrent PGM.** Nucleotide variants were subdivided in two frequency classes: high (frequency minor allele > 15%, n = 4) and low (frequency minor allele: < 15%, n = 42). Mean proportions ± s.e. of the minor variants detected in PR8 and PR8mut viral samples by the Illumina MiSeq and Ion Torrent PGM are shown. Minor allele proportions were analyzed by logistic regression (link function = logit). Significance levels of pairwise comparisons were assessed by a Fisher’s protected least significance difference test * = p < 0.05, ** = p < 0.01.

The average difference between the frequencies of a variant in PR8 sequencing duplicates was only 0.17% ± 0.12% for the Illumina MiSeq and 0.16% ± 0.18% for the Ion Torrent PGM, again indicating that both sequencing platforms provide reproducible output (Table 8). However, the frequency of occurrence of the variants differed substantially between sequencers. For example, the mean variant frequency differed between 0.06% (position 1199 in the PR8 HA segment) and 4.5% (position 1249 in the PR8 NP segment) for the same viral sample sequenced on both sequencers. In addition, most detected variants were present at a lower frequency based on the Ion Torrent PGM output. Similar results were obtained for the PR8mut samples (Table 9). To determine whether this difference in frequencies is significant between the sequencing platforms, variant frequencies obtained in PR8 and PR8mut were analyzed using logistic regression, considering loci with low (< 15%) and high (> 15%) minor variant frequencies as separate classes. This analysis clearly indicates that when the minor variant is present at a low frequency, the Illumina MiSeq systematically detects the minor variants at significantly higher frequencies than the Ion Torrent PGM (Figure 10).

**Table 9:** **Mutant PR8 quasispecies sequenced in duplicate on both Illumina MiSeq and Ion Torrent PGM**

						**Frequency (in %)**				
**Segment**	**Position**	**Type**	**Reference**	**Allele**	**aa change**	**Illumina MiSeq**		**Ion Torrent PGM**		**Function/location**
PB2	416	SNP	A	G	silent	1.55	1.30	0.59	0.57	/
**PB1**	**1486**	**SNP**	**A**	**G**	**Lys481Arg**	2.52	2.80	1.79	2.37	K481 crucial for polymerase function *in vivo*, not *in vitro* [47]
PA	212	SNP	G	T	Glu56Asp	5.72	4.85	2.09	2.18	located in endonuclease domain [58,59]
PA	1139	SNP	G	T	Gln365His	2.50	2.40	1.00	1.05	located in PB1 interacting domain [60]
HA	524	SNP	A	C	Ser158Arg	13.17	12.93	9.70	9.63	Compensatory mutation in [61], located in Ca antigenic site [54]
HA	524	SNP	A	T	Ser158Cys	0.98	0.90	0.61	0.63	located in variable Ca antigenic site [54]
**HA**	**607**	**SNP**	**A**	**G**	**silent**	1.54	1.43	2.23	2.47	/
**HA**	**747**	**SNP**	**A**	**G**	**Glu232Gly**	39.95	40.14	36.74	36.02	receptor specificity [50]
**HA**	**765**	**SNP**	**A**	**G**	**Asp238Gly**	3.17	3.07	1.50	1.44	enables binding to α2,3- and α2,6-linked sialic acids [52,53]
**HA**	**823**	**SNP**	**A**	**G**	**Ile257Met**	2.92	3.08	1.49	1.64	located in head domain close to Sa antigenic site [54]
HA	828	SNP	A	G	Glu259Gly	5.17	4.96	2.09	1.95	located on surface head domain close to Sa antigenic site [54]
HA	1088	SNP	T	A	Phe346Ile	6.99	6.76	3.75	4.18	located in fusion peptide [62]
HA	1090	SNP	T	G	Phe346Leu	1.28	1.09	0.59	0.69	located in fusion peptide [62,63]
HA	1109	SNP	A	G	Ile353Val	59.69	60.02	62.48	61.74	described as fusion peptide pseudorevertant [62,64]
**HA** ^**#**^	**1199**	**SNP**	**A**	**G**	**Ser383Gly**	1.13	1.18	0.97	1.05	located in stem domain
**HA**	**1330**	**SNP**	**A**	**G**	**silent**	1.59	1.67	1.94	1.14	/
HA	1424	SNP	G	T	Val458Leu	3.43	3.13	1.63	1.64	located in stem domain, not surface exposed
HA	1430	SNP	A	G	Asn460Asp	10.08	9.60	5.82	6.10	present in the PR8 quasispecies grown on MDCK cells [65]
HA	1431	SNP	A	G	Asn460Ser	14.79	14.62	10.15	10.29	located in stem domain
HA	1487	SNP	G	A	Gly479Arg	1.59	1.39	0.61	0.57	located in stem domain, not surface exposed
NP	635	SNP	G	A	silent	4.44	3.93	2.45	1.87	/
NP^#^	739	SNP	T	C	Ile225Thr	1.11	1.15	0.52	0.56	surface exposed, in NP-NP interaction domain [56]
NA	476	SNP	T	A	Cys146Ser	6.85	6.71	4.33	3.85	located in head domain, involved in coupling of subunits [66]
NA	994	SNP	C	T	silent	1.03	1.00	0.60	0.65	/
M	354	SNP	C	T	introduced	99.96	99.96	99.61	99.62	/
M	645	SNP	A	T	introduced	99.95	99.95	99.95	99.95	/
NS	409	SNP	G	T	NS1: Gln121His	40.37	39.54	31.96	32.61	situated next to the NS1_122–130_ CTL epitope [67]
NS	549	SNP	G	A	NS1: Gly168Glu	1.27	1.20	0.71	0.59	NS1: located in effector domain [68]
										NS2: N-terminal domain
					NS2: Asp11Asn					
NS^#^	564	SNP	A	G	NS1: Asp173Gly	1.05	1.10	0.65	0.64	NS1: located in effector domain [68]
					NS2: Met16Val					NS2: Met16 is involved in nuclear export NP [69]

^#^not present in Genbank or Influenza Research Database.

Bold = variants also present in PR8 quasispecies.

HA segment = numbering of HA amino acid residues is based on the PR8 HA open reading frame with the starting methionine as position = 1.

Almost all mutations detected in the wild type and/or mutant PR8 quasispecies are also present in H1N1 viral sequences retrieved from the Influenza Research Database and/or Genbank. The exceptions are indicated with a number sign (#) in Tables 8 and 9. These sequence variants (Glu203Lys and Ser383Gly in HA, Ile225Thr in NP and Asp173Gly/Met16Val in NS1/NS2) might exist in nature but have not been reported yet. Most of the detected mutations are present in the HA segment, which is also the most variable influenza protein in nature [70]. Most of the detected mutations were substitutions occurring at a frequency < 5%. However, three mutations in HA and one in NP of PR8 as well as four mutations in HA and one in NS of PR8mut were present at a frequency > 10% (based on Illumina MiSeq data) (Tables 8 and 9). Of all detected variants, only seven (five non-synonymous and two synonymous) were shared by both PR8 and PR8mut and present in all samples sequenced. These were all in the HA segment, except for one variant in PB1 (Tables 8 and 9, bold).

Taken together, these results show that both the wild type and mutant PR8 virus behave as a fairly heterogeneous virus populations even in the absence of external selection pressure.

## Discussion

Next generation sequencing (NGS) has become increasingly valuable to study virus diversity. NGS instruments have a very high sequencing capacity and therefore allow a very high coverage of the relatively small genome of RNA viruses. NGS analysis is thus in principle well suited for determining the genetic heterogeneity of RNA viruses. Unfortunately, in many research articles on viral quasispecies diversity there is little information on how the raw data were processed. Furthermore, the performance of different commercially available NGS platforms for quasispecies analysis has not been evaluated. Here, we compared the quality of the sequencing output obtained on the Illumina MiSeq and Ion Torrent PGM benchtop sequencers. We also propose an analysis pipeline for *in silico* processing of the sequencing data that allows identification and frequency determination of nucleotide variants in the influenza A virus (Figure 3). This analysis pipeline will help to standardize variant calling in small RNA genomes based on NGS data.

To determine the influenza genome diversity by NGS technology, different hurdles have to be overcome. First, it is technically challenging to obtain high quality full-length RT-PCR products that cover the complete segmented RNA genome of influenza viruses. We optimized an RT-PCR protocol with primers based on the conserved 3′ (Uni12) and 5′ ends (Uni13) of the eight genome segments [42,43,71]. Critical steps in this protocol are primer concentration and annealing and elongation times. Because the sequence of these segment ends is conserved, this RT-PCR should be applicable to different influenza A virus strains.

A second hurdle is to distinguish between mutations that truly represent the viral genome diversity from errors introduced by RT-PCR amplification and the NGS chemistry. The first step is to filter the output sequence data *in silico* to retain only high quality reads. However, the available software and filtering parameters vary and are not always clearly described in the literature, making comparison of results very difficult. To reduce false positive variant calls introduced by the sequencing method, we applied specific trimming, filtering and variant calling parameters in the CLC Genomics Workbench software. We first applied this bioinformatics analysis pipeline to sequencing reads derived from plasmid DNA samples. We removed adaptor contamination, ambiguous nucleotides and trimmed low quality bases at the end of the reads by applying a Phred score of 20. Then, we excluded reads shorter than 50 bases to avoid unspecific mapping of these short reads. Trimming eliminated relatively more bases from the Ion Torrent PGM, meaning that the base quality of sequencing reads from the Ion Torrent PGM is lower than that from the Illumina MiSeq. In other words, the potential advantage of longer read lengths obtained with the Ion Torrent machine was cancelled by their relatively low quality. Together this resulted in a higher relative loss of bases for the Ion Torrent PGM data than for the Illumina MiSeq data (21.01% versus 14.01% respectively). Furthermore, the Phred score distribution across the reads, a measure of the intrinsic sequencing quality, was higher for the Illumina MiSeq data than for the Ion Torrent PGM data, resulting in a lower error rate. After this quality control, the sequencing reads were mapped to the reference sequence, resulting in a higher percentage of mapped reads for the Illumina MiSeq. The total mapping error rate of the Illumina MiSeq (mainly nucleotide substitutions) was lower than that of the Ion Torrent PGM (mainly indels). This finding is in agreement with Loman and colleagues [20]. However, for plasmid DNA analysis the substitution error rate on the Ion Torrent PGM appeared to be lower than that of Illumina MiSeq (Figure 5). After variant calling, the resulting hits were filtered based on frequency, forward/reverse balance, average quality, and independent counts to remove false positive variants. After filtering, both sequencers detected the tracer mutations we had introduced with excellent accuracy and sensitivity. Nevertheless, the average quality (Phred score) of the detected variants was higher on the Illumina MiSeq than on the Ion Torrent PGM, making the variants detected on the Illumina MiSeq more reliable. The number of false positive variants can be further reduced by cross-platform replication, but the different biases of the sequencing platforms may cause many true variants to be overlooked when cross-platform replicates are compared [72,73].

We then applied the analysis pipeline outlined in Figure 3 to PR8 and PR8mut virus, which were generated by a plasmid-based reverse genetics system and amplified in MDCK cells. In our opinion, variants in the influenza virus genome that appear with a frequency below 0.5% are very difficult to distinguish from the background noise that is cumulatively introduced by RT-PCR and the inherent variation due to the chemistry of currently available Illumina and Ion Torrent sequencers. We propose that a similar threshold of 0.5% should be applied to interpret the genetic diversity of RNA viruses. Nevertheless, mutations with a frequency as low as 0.05 − 0.2% in Chikungunya virus have been reported in the literature as meaningful based on Illumina GAIIX sequencing [74]. Given the error rate of the influenza virus polymerase, resulting in approximately one mutation per 10.000 nucleotides, together with the errors introduced during RT-PCR and the technical background error rate of the NGS platforms applied in this study, it is not straightforward for both the Illumina MiSeq and the Ion Torrent PGM to identify each variant in the viral quasispecies. Nevertheless, even with the threshold of 0.5% proposed here, NGS will enable studying of the viral diversity in much more detail than in the past.

Our analysis showed that the *de novo* assembled PR8 and PR8mut sequences correspond very well to the plasmid-derived reference genome. We detected 19 mutations in PR8 and 29 mutations (including the two tracer mutations) in PR8mut with a frequency of 0.5% or higher. When a variant was present at low frequency (< 15%), the Illumina MiSeq detected it with significantly higher frequency than the Ion Torrent PGM. Most of the detected mutations were transitions and appeared with a frequency below 5%. However, three mutations in HA and one in NP of PR8, as well as four mutations in HA and one in NS of PR8mut, were present at a frequency > 10% (based on Illumina MiSeq data) (Tables 8 and 9). We detected only one single nucleotide deletion in the PR8 virus. This deletion was in a homopolymer at position 1482 in PB1 but was detected with a frequency of 2 − 3% by both sequencers, in both duplicates of PR8 virus. In addition, this deletion was also detected with a similar frequency in both PR8mut samples sequenced on the Illumina MiSeq and in one of the duplicate samples sequenced on the Ion Torrent PGM. This deletion disrupts the open reading frame, leading to premature termination of PB1. This detrimental mutation is in line with the finding of Brooke and colleagues, who showed that most of the infectious influenza A virions fail to express detectable levels of one or more viral proteins [75].

**Figure 11:**
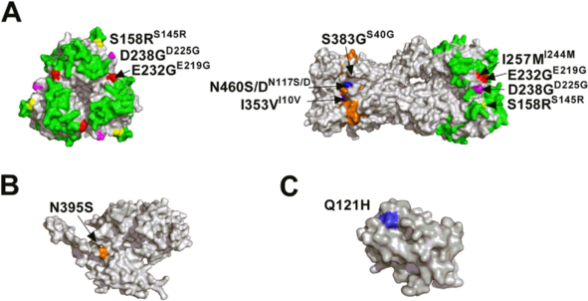
**Position of variants present in PR8 and PR8mut quasispecies in the HA, NP and NS1.** The variants in HA (hemagglutinin), NP (nucleoprotein) and NS1 (non-structural protein 1) detected in the PR8 and PR8mut quasispecies were modeled with PyMol (Delano Scientific, http://www.pymol.org), using the HA from A/Puerto Rico/8/1934 (H1N1) (PDB code: 1RVX), the NP from A/Wilson-Smith/1933 (H1N1) (PDB code: 2IQH) and the effector domain of NS1 from A/Puerto Rico/8/1934 (H1N1) (PDB code: 3RVC). **(A)** Top (left) and lateral (right) view of the surface exposed amino acids of the HA trimer. The Cb, Ca, Sa and Sb antigenic sites are shown in green. The mutations that are present in both PR8 and PR8mut are shown in red or in magenta if they overlap with the antigenic sites. Mutations in PR8mut that are present at a frequency > 5% are shown in blue or in yellow when overlapping with the antigenic sites or in purple when overlapping with the fusion peptide (orange). The mature H3 amino acid numbering of the variants is provided in superscript. **(B)** Lateral view of the NP monomer with the N395S mutation present in PR8 shown in brown. **(C)** The effector domain of NS1 with the Q121H mutation in PR8mut shown in blue.

We focused on the mutations detected by both sequencers with a frequency > 5% and on the mutations that appeared in both wild type and mutant PR8 viruses. There are three such mutations in the HA head domain of PR8 and four in the HA head domain of PR8mut, and all of them are part of or close to the antigenic sites (Figure 11A). The shared Asp238Gly mutation (Asp225Gly for H3 numbering) is associated with enhanced virion binding to the avian-type Sia(α2-3)Gal and was reported previously as a position that is selected by egg-adaptation of influenza viruses [76]. The Ser158Arg mutation (Ser145Arg for H3 numbering) in PR8mut has been described as a compensatory mutation in PR8 virus possessing the Lys165Glu mutation in HA (H3 numbering), which decreases the receptor binding avidity and replication kinetics of the virus [61]. The two mutations in the stem domain are relatively conservative (Ser383Gly and Val458Met; Ser40Gly and Val115Met for H3 numbering of HA2) and therefore might not affect virus replication. Remarkably, the G-to-A substitution at position 1424, leading to the Val458Met change in HA, had a frequency close to 100% in the PR8 HA segment but was absent in PR8mut (although a Val458Leu change is present in a small percentage of PR8mut). This mutation was probably fixed in the wild type virus genome at a very early step, *e.g.* during plaque purification of the PR8 seed virus we used to prepare stock virus. We also picked up two other codon changes in the HA stem region of PR8mut: Asn460Asp (5 − 10%) and Asn460Ser (10 − 15%) (Asn117Asp and Asn117Ser for H3 numbering of HA2). Based on pyrosequencing of the HA segment, the Asn460Asp mutation has been observed in 12.2% in PR8 virus grown on MDCK cells [65]. In addition, the PR8mut caries the Ile353Val (Ile10Val for H3 numbering of HA2) mutation in the HA fusion peptide at a frequency of about 60%. A valine at this position has been observed in a PR8 pseudo-revertant after introducing the Ile10Ala mutation. A valine at this position is compatible with the α-helical structure of the fusion peptide [64]. Both PR8 viruses also contain mutations in other segments. For example, both viruses share the conservative Lys481Arg mutation in PB1. This lysine at position 481 is crucial for the polymerase function of PB1 *in vivo* but mutating it to alanine was tolerated *in vitro* [47]. In wild type PR8, the Asn395Ser variant in NP is in a domain involved in NP − NP and NP − PB2 interactions (Figure 11B) [77]. The Gln121His variant detected in NS1 of PR8mut is situated just before a human CTL epitope (Figure 11C) [67]. Remarkably, none of the variants we observed correspond to the variants described in an earlier study, in which a PR8 strain (originally adapted for growth on embryonated chicken eggs) was adapted for growth on MDCK cells [78]. However, we used MDCK cells only to expand our virus stock, which corresponds to about six cycles of PR8 virus replication. Furthermore, we generated our PR8 virus starting from eight plasmids, indicating that the passaging history is a determinant of the variants detected in an influenza virus quasispecies.

Both sequencers are highly effective for accurate detection of low frequency mutations, but each one has its advantages and limitations. On the one hand, the Illumina MiSeq platform has about three times higher output capacity than the Ion Torrent PGM, enabling sequencing of more samples in parallel on the Illumina MiSeq. On the other hand, the Ion Torrent PGM is significantly faster: its time from sample preparation to data analysis is one day less than for the Illumina MiSeq. After the *in silico* quality control, the two sequencers produced reads of comparable lengths. The Illumina MiSeq had a higher intrinsic sequencing quality than the Ion Torrent PGM, presumably because detecting incorporated bases based on a coupled fluorescent dye (Illumina) gives less noise than a change in pH caused by release of a proton after incorporation of a base (Ion Torrent). However, the Ion Torrent PGM had a lower false-positive rate for detecting SNPs. Another interesting observation is the lower coverage of the ends of the viral segments on the Illumina MiSeq due to the transposase-based fragmentation. Nextera transposase-based fragment library preparation is convenient and fast but results in low coverage of segment termini. We also noticed some sequence bias of this transposase-based fragmentation approach (Figures 8 and 9). Mechanical fragmentation followed by adaptor ligation enables comparable coverage of all bases of the influenza virus genome, and is therefore the preferred method for library preparation (Figures 8 and 9).

The proposed RT-PCR protocol and subsequent analysis pipeline for influenza viruses is widely applicable, *e.g.* to study vaccine composition, analyze virus evolution under selection pressure, monitor mutations associated with antiviral resistance, and assemble the reference genome of new viral isolates. For clinical samples, the shorter turnaround time of the Ion Torrent PGM (sample preparation, sequencing and analysis in about 2 days) is clearly advantageous to the Illumina MiSeq (about 3 days). In contrast, when analyzing many viral samples at high coverage, the greater output of the Illumina MiSeq is an important advantage.

## Conclusion

Our study underlines the power and limitations of two commonly used next-generation sequencers for the analysis of influenza gene diversity. We propose an *in silico* pipeline for selecting high quality reads obtained by NGS platforms. This pipeline is also more widely applicable. Due to the lower total error rate and the higher sequencing quality of the reads, we conclude that the Illumina MiSeq platform is more suited than the Ion Torrent PGM for detecting variant sequences, whereas the Ion Torrent platform has a shorter turnaround time. In addition, we found that the detection limit for reliable recognition of variants in the viral genome required a frequency of 0.5% or higher.

## Methods

### Cell lines

MDCK and HEK293T cells were cultured in Dulbecco’s Modified Eagle medium (DMEM) supplemented with 10% fetal calf serum, non-essential amino acids, 2 mM L-glutamine, 0.4 mM sodium pyruvate, 100 U/ml penicillin and 0.1 mg/ml streptomycin at 37°C in 5% CO_2_.

### Generation and production of plasmids with tracer mutations

Reverse genetics plasmids for PR8 virus were kindly provided by Dr. Robert G. Webster (St. Jude Children’s Research Hospital, Memphis, USA) [31]. We introduced two silent mutations in the M coding gene, a C-to-T substitution at position 797 (numbering relative to the human cytomegalovirus promoter in the pHW197-M plasmid) and an A-to-T substitution at position 1088 in pHW197-M. These two positions were selected as follows. First, we generated a consensus sequence of the M-gene based on all full-length segment 7 sequences of human H1N1 viruses present in the Influenza Virus Resource Database (NCBI) on September 11th, 2011. Next, we aligned the consensus sequence to the M segment of PR8 (present in pHW197-M) and selected two synonymous mutations in the M1 open reading frame at positions C354T and A645T (segment 7 numbering). These two mutations were introduced by two consecutive rounds of quickchange site-directed mutagenesis (Stratagene) at positions C797T and A1088T in pHW197-M to generate pHW197-Mmut. The two mutations also introduced a HindIII and a PvuII restriction site, respectively. These plasmids and the plasmids encoding the other seven PR8 genome segments were transformed and amplified in *E. coli* DH5α. Plasmid DNA was isolated with the Plasmid Midi Kit (Qiagen) according to the manufacturer’s instructions. The resulting air-dried pellet was dissolved in 50 μl of sterile ultrapure water. The presence of the introduced mutations in pHW197-Mmut was confirmed by restriction analysis and Sanger sequencing on a capillary sequencer (Applied Biosystems 3730XL DNA Analyzer).

### Generation of recombinant PR8 and PR8mut viruses

To generate recombinant wild type PR8 virus and PR8 virus with the two tracer mutations in the M gene (PR8mut), 1 μg of pHW191-PB2, pHW192-PB1, pHW193-PA, pHW194-HA, pHW195-NP, pHW196-NA and pHW198-NS, together with 1 μg of pHW197-M (wild type PR8) or pHW197-Mmut (PR8mut) was transfected using calcium phosphate co-precipitation into a HEK293T-MDCK cell co-culture in Opti-MEM (3 × 10^5^ HEK293T and 2 × 10^5^ MDCK cells in a 6-well plate). After 30 h, L-1-tosylamide-2-phenylethyl chloromethyl ketone (TPCK)-treated trypsin (Sigma) was added to a final concentration of 2 μg/ml. After 72 h, the culture medium was collected and the presence of virus was confirmed by hemagglutination of chicken red blood cells. Reverse genetics-generated PR8 and PR8mut viruses were plaque-purified on MDCK cells as follows. Confluent MDCK cells in a six-well plate were infected with a serial dilution series of virus. After 1 h, an overlay of low melting agarose (Type VII agarose, Sigma; final concentration 1%) in serum-free cell culture medium containing 2 μg/ml TPCK-treated trypsin (Sigma) was added. After 56 h, cytopathic effect was checked, agar overlaying viral plaques were selected with a pipette tip, and virus was allowed to diffuse from the agar for 24 h at 4°C in serum-free medium. Afterwards, virus derived from one plaque was amplified on MDCK cells in serum-free cell culture medium in the presence of 2 μg/ml TPCK-treated trypsin (Sigma). After 96 h, the culture medium was collected, and cell debris was removed by centrifugation for 10 min at 2500 g at 4°C, and the virus was pelleted from the supernatants by overnight centrifugation at 16,000 g at 4°C. The pellet was dissolved in sterile 20% glycerol in PBS, aliquoted and stored at −80°C. The infectious titer of the obtained PR8 and PR8mut virus stocks was determined by plaque assay on MDCK cells, on three different aliquots each performed in triplicate. The presence of the introduced mutations in the M segment of PR8mut was confirmed by segment-7-specific RT-PCR followed by purification from 1% agarose gel (High Pure PCR Product Purification Kit, Roche) and conventional Sanger sequencing of the amplified PCR fragment.

### Plaque assay

MDCK cells were seeded in complete DMEM in 12-well plates at 3 × 10^5^ cells per well. After 18 h, the cells were washed once with serum-free medium and incubated (in triplicate) with a two-fold dilution series of the virus (made in serum-free cell culture medium containing 0.1% BSA) in 500 μl medium. After 1 h incubation at 37°C, an overlay of 500 μl of 1.6% Avicel RC-591 (FMC Biopolymer) in serum-free medium with 4 μg/ml TPCK-treated trypsin (Sigma) was added. After incubation at 37°C for 48 h, the overlay was removed and the cells were fixed with 4% paraformaldehyde and permeabilized with 20 mM glycine and 0.5% (v/v) Triton X-100. Plaques were stained with an anti-M2e IgG1 mouse monoclonal antibody (final concentration 0.5 μg/ml) followed by a secondary anti-mouse IgG horseradish peroxidase (HRP)-linked antibody (GE Healthcare). After washing, TrueBlue peroxidase substrate (KPL) was used to visualize the plaques.

### RNA isolation

RNA was isolated with the High Pure RNA Isolation Kit (Roche) according to the manufacturer’s instructions, excluding the DNase I digestion step. In brief, a 200-μl sample containing 1 x 10^7^ PFU of stock virus in serum-free cell culture medium with 0.1% BSA was combined with 400 μl lysis-binding buffer and mixed by vortexing. The mixture was loaded on a two-layered glass fiber column. After binding to the column and washing, the RNA was eluted in 50 μl elution buffer (water, PCR grade).

### RT-PCR

Primers used for cDNA synthesis and PCR were designed based on the 5′ and 3′ conserved ends of the influenza A genomic segments and contain an additional sequence of 20 nucleotides at their 5′ end necessary for PCR amplification [41-43,79]. cDNA was generated using the Transcriptor First Strand cDNA Synthesis Kit (Roche). Reverse transcription was performed with the Transcriptor Reverse Transcriptase (10 U, Roche), using 12.5 μl RNA, 2.5 μM CommonUni12G primer (GCCGGAGCTCTGCAGATATCAGCGAAAGCAGG), 1x Transcriptor Reverse Transcriptase Reaction Buffer, 20 U Protector RNAse inhibitor and 4 mM dNTPs, in a total volume of 20 μl. The components were mixed, and the reaction was incubated for 15 min at 42°C, 15 min at 55°C, 5 min at 60°C, and finally 5 min at 85°C to inactivate the reverse transcriptase. Ten microliters of the resulting cDNA sample was amplified in a 100-μl PCR reaction using 2 U Phusion High Fidelity polymerase (Thermo Scientific), 0.2 μM CommonUni12G and CommonUni13 (GCCGGAGCTCTGCAGATATCAGTAGAAACAAGG), 0.2 mM dNTPs, and 1× High-Fidelity buffer. Thermocycling was performed in a PTC-200 Thermal Cycler (MJ Research) with the following conditions: initial denaturation for 30 s at 98°C, 25 cycles of 10 s at 98°C followed by 7.5 min at 72°C, and a final elongation step of 7 min at 72°C. PCR products were purified using the High Pure PCR Product Purification kit (Roche) according to the manufacturer’s instructions, and the product was eluted in 50 μl sterile ultrapure water (preheated to 65°C). One microgram of the product was analyzed by agarose gel electrophoresis (1.5% agarose gel) followed by ethidium bromide staining.

### Illumina MiSeq sequence determination

We used 0.5 ng of purified plasmid or RT-PCR sample and the Nextera XT DNA Sample Preparation Kit (Illumina) according to the manufacturer’s instructions to generate multiplexed paired-end sequencing libraries. Sequencing libraries were generated in duplicate, meaning that from each plasmid or RT-PCR sample two libraries were prepared in parallel and sequenced on the same Illumina MiSeq sequencing chip. In brief, DNA samples were fragmented and tagged with adapters by Nextera XT transposase. These adaptor ligated DNA fragments were amplified by a limited-cycle PCR program (12 cycles) to add the barcodes and sequences required for subsequent cluster formation. The resulting fragments were purified and simultaneously size-selected by using 0.6× AMpure beads. Fragments were analyzed on a High Sensitivity DNA Chip on the Bioanalyzer (Agilent Technologies) before loading on the sequencing chip. The fragment lengths showed a negatively skewed distribution with a peak at approximately 700–1000 bases. From the optimized RT-PCR products, also 500 ng was sheared with an M220 focused-ultrasonicator (Covaris) set to obtain peak fragment lengths of 300–400 bp. Next, the NEBNext Ultra DNA Library Preparation kit (New England Biolabs) was used to repair the ends and to add Illumina MiSeq-compatible barcode adapters to 100 ng of fragmented DNA. The resulting fragments were size-selected using Agencourt AMPure XP bead sizing (Beckman Coulter). Afterwards, indexes were added in a limited-cycle PCR (10 cycles), followed by purification on Agencourt AMpure XP beads. Fragments were analyzed on a High Sensitivity DNA Chip on the Bioanalyzer (Agilent Technologies) before loading on the sequencing chip. Equimolar amounts of normalized libraries were combined and diluted 25-fold in hybridization buffer. The multiplex sample was heat denatured for 2 min at 96°C before loading on the MiSeq chip. After the 2×250 bp MiSeq paired-end sequencing run, the data were base called and reads with the same barcode were collected and assigned to a sample on the instrument, which generated Illumina FASTQ files (Phred +64 encoding). These files were imported in the CLC Genomics Workbench software (CLC Bio, Qiagen). During import in CLC Genomics Workbench, the uncallable ends of the MiSeq reads (B in input file) were automatically trimmed and the failed reads (Y in header information for the quality score) were removed.

### Ion Torrent PGM 318 chip sequence determination

Samples for sequence analysis were generated in duplicate, meaning that from each plasmid or RT-PCR sample two libraries were prepared in parallel for sequencing on the same Ion Torrent PGM 318 sequencing chip. From each plasmid or RT-PCR product, 100 ng was sheared with an M220 focused-ultrasonicator (Covaris) set to obtain peak fragment lengths of 400–500 bp. After shearing, blunt ends were created using the end repair enzyme from the Ion Plus Fragment Library kit (Life Technologies). Next, the fragments were ligated to Ion Torrent PGM-compatible barcode adapters. Since the adaptors are not 5′ phosphorylated, the nick repair polymerase in the kit repairs subsequently the nick on one strand at each ligation site, in order to minimize adaptor-dimer formation. We purified and simultaneously size-selected the adapter-ligated library using Agencourt AMPure XP bead sizing (Beckman Coulter). Fragments were analyzed on a High Sensitivity DNA Chip on the Bioanalyzer (Agilent Technologies); the fragment length peak was situated around 450 bp. Barcoded libraries were pooled in equimolar amounts. From the resulting diluted multiplexed library, 20 μl was loaded on an Ion OneTouch 2 instrument (Life Technologies) to perform emulsion PCR on Ion Sphere particles using the Ion PGM Template OT2 400 kit. We used the Ion PGM sequencing 400 kit (Life Technologies) to sequence templated ion sphere particles deposited in the Ion 318 chip v2 (revision 2.0, Life Technologies). The Ion Torrent Suite version 4.6 (Life Technologies) was used with the default parameters for base calling and assigning of the reads to a sample based on their barcode. The default settings in the Ion Torrent Suite already filter and trim the sequencing reads to some extent. These default trimming parameters are not stringent and remove only very low quality 3′ ends (mean Phred score of at least 15 in a base window of 30) and adaptor contamination. The resulting FASTQ files were imported into CLC Genomics Workbench for further analysis.

### Analysis of sequencing data

CLC Genomics Workbench version 7.0.3 (CLC Bio, Qiagen) was used to analyze and process the sequencing reads of both the Ion Torrent PGM and the Illumina MiSeq. First, adaptor contamination was removed from the reads. Next, the sequencing reads were trimmed from both sides using the modified Mott trimming algorithm to reach a Q20 score, which means that the chance that a particular base in the sequence is called incorrectly by the sequencer is 1 in 100. Afterwards, all ambiguous (N) bases were trimmed from the reads. We also removed the reads with a read length below 50. For the Illumina MiSeq, the broken pairs resulting from trimming and filtering were also removed. The remaining reads were assembled using default settings for *de novo* assembly. In addition, the processed reads were also aligned with the pHW197-M plasmid reference sequence or the influenza PR8 reference genome (based on the sequences encoding the eight segments in the pHW vectors, determined by Sanger sequencing, with addition of the extra 20 nucleotides present at the 5′ site in the RT-PCR primers) using local alignment. For this, the following default penalties were used: match = +1, mismatch = −2, insertion/deletion = −3, filtering threshold: length fraction = 0.9 and similarity fraction = 0.8. Non-specific matches, defined as reads aligning to more than one position with an equally good score, were ignored. Sequence variants were called using all available sequencing data that covered each nucleotide at least 100 times and had a central base quality score of Q20 or greater. The A-to-G variant introduced by the primer at position 24 in the HA, NP, NA, M and NS segments was not taken into account during the influenza quasispecies variant analysis. All numerical data mentioned in the text are presented as averages with their standard deviations (± SD).

### Statistical analysis

Sequence variants with the lowest proportion were considered as minor alleles. Analysis of minor allele proportions was performed by fitting a logistic regression model of the form logit(p) = constant + PLATFORM*VIRUS*CLASS + error, where p indicates the minor allele proportion, PLATFORM refers to the sequencing platform, VIRUS refers to virus population, and CLASS refers to class of loci having either low (< 15%) or high (> 15%) minor variant frequencies. Significance of the fixed PLATFORM, VIRUS and CLASS effects was assessed by an F-test. Significance of pair-wise comparisons between mean proportions was assessed by a Fisher’s protected least significance difference test. The logistic regression and assessment of significance was performed in Genstat v16.

### Sequencing data

The output sequencing reads obtained on the Illumina MiSeq and Ion Torrent PGM were submitted to NCBI’s Sequence Read Archive and can be found under project numbers SRP052608 (plasmid samples) and SRP052225 (viral samples).
